# Exploring the Effect of Nutrition on Fetal Development in Pregnant Patients With Inflammatory Gut Diseases: A Scoping Review

**DOI:** 10.7759/cureus.115649

**Published:** 2026-09-02

**Authors:** Zena K Moody, Anitha T Joseph, Ravdeep Gill, Rajin Persaud, Adriana Gurreri, Christo Manikkuttiyil, Adina Ahmed, Shreya Golakoti, Sarah Villamil, Ravi Ghotra, Ioana Scripa

**Affiliations:** 1 College of Osteopathic Medicine, Nova Southeastern University Dr. Kiran C. Patel College of Osteopathic Medicine, Miami, USA; 2 College of Osteopathic Medicine, Nova Southeastern University Dr. Kiran C. Patel College of Osteopathic Medicine, Fort Lauderdale, USA; 3 College of Osteopathic Medicine, Nova Southeastern University Dr. Kiran C. Patel College of Osteopathic Medicine, Davie, USA; 4 Nutrition, Nova Southeastern University Dr. Kiran C. Patel College of Osteopathic Medicine, Fort Lauderdale, USA

**Keywords:** diet restriction, fetus development, inflammatory bowel disease, nutritional deficiency, pregnancy, vitamin supplementation

## Abstract

Inflammatory gut diseases (IGDs) are increasing in prevalence and pose unique challenges during pregnancy. Poorly controlled IGDs in pregnant women are linked to adverse fetal outcomes such as low birth weight, preterm birth, and impaired gut microbiome development. Nutritional deficiencies, particularly in magnesium, folate, and vitamin B12, exacerbate these risks due to both inadequate intake and malabsorption. This scoping review aimed to evaluate the role of nutrition in fetal development among pregnant women with IGDs, highlighting dietary strategies that may enhance maternal gastrointestinal health and optimize fetal outcomes. This study was designed as a scoping review to examine the relationship between IGDs, pregnancy, and nutrition. Following the Preferred Reporting Items for Systematic Reviews and Meta-Analyses extension for scoping review (PRISMA-ScR) guidelines, a search was conducted for peer-reviewed literature using the databases Embase, Web of Science, Ovid MEDLINE, and CINAHL. The search was restricted to studies published in English within the last ten years and that focused on pregnant or postpartum patients and fetuses in the context of nutrition and IGDs. The search strategy used predefined terms in multiple combinations with Boolean operators: “inflammatory bowel disease”, “pregnancy”, “fetus development”, “nutritional deficiency”, “diet restriction”, and “vitamin supplementation”. The search identified 1,589 records, of which 728 remained after duplicate removal. Following title and abstract screening, 103 full-text reports were sought for retrieval, 98 were assessed for eligibility, and five studies met the final inclusion criteria. The included studies suggest that multidisciplinary antenatal care, maintenance of disease remission, and attention to maternal nutritional status may support favorable maternal and neonatal outcomes. However, the available evidence is limited, consists primarily of observational studies, and no included study directly evaluated nutritional interventions and fetal outcomes. Research on fetal outcomes remains limited and warrants future investigations exploring long-term fetal outcomes, the impact of maternal nutritional deficiencies, and dietary interventions to improve treatment protocols. Advancements in this research can ultimately improve maternal and neonatal outcomes in IGD-affected pregnancies.

## Introduction and background

Inflammatory gut diseases (IGDs) are a group of chronic inflammatory disorders affecting the gastrointestinal tract and include inflammatory bowel diseases (IBD), such as Crohn’s disease (CD) and ulcerative colitis (UC), as well as other immune-mediated conditions such as celiac disease [1-4]. CD is characterized by transmural gastrointestinal inflammation with skip lesions, whereas UC primarily affects the rectum and colon with continuous superficial inflammation [2,3]. These conditions may impair nutrient absorption and nutritional status, making their management during pregnancy particularly important for maternal and fetal health. Although this review was designed to evaluate IGDs broadly, all studies meeting the eligibility criteria focused exclusively on IBD, highlighting an important gap in the literature regarding other IGDs during pregnancy.

This review focuses on pregnant women with IGDs and the role of maternal nutrition in fetal development. Adequate maternal nutrition before and during pregnancy is essential for healthy fetal growth and development [5,6]. Deficiencies in key micronutrients, including magnesium, folate, and vitamin B12, have been associated with adverse pregnancy outcomes such as fetal growth restriction, preterm birth, and neural tube defects [5,7,8]. Because IBD is frequently associated with chronic intestinal inflammation and malabsorption, pregnant women with these conditions are at increased risk for nutritional deficiencies that may adversely affect maternal and fetal health.

Active IBD during pregnancy is associated with adverse outcomes, including low birth weight and preterm birth [9,10]. Nutritional deficiencies, including vitamin D, vitamin B12, calcium, iron, zinc, and folate, may further complicate pregnancy in women with IBD because of inadequate intake and impaired intestinal absorption [9]. Alterations in the maternal and prenatal gut microbiome may also influence offspring development through microbiome-associated metabolic and epigenetic pathways [11]. Although several dietary components have demonstrated effects on intestinal barrier function in non-pregnant patients with IBD [12], their effects during pregnancy and on fetal outcomes remain unclear.

Maintenance of disease remission during pregnancy is associated with improved maternal and neonatal outcomes in women with IBD. Dietary modification, including a low-fermentable oligosaccharides, disaccharides, monosaccharides, and polyols (low-FODMAP) diet and individualized dietary approaches, has emerged as an important adjunct to disease management and may improve gastrointestinal symptoms and overall nutritional status [13-15]. However, evidence evaluating the effectiveness of these dietary strategies during pregnancy and their direct influence on fetal outcomes remains limited [16,17].

Although previous studies have demonstrated an increased risk of adverse pregnancy and neonatal outcomes among women with IBD, evidence-based nutritional recommendations specific to this population remain poorly defined [18]. Existing literature has largely focused on disease activity and obstetric outcomes rather than identifying dietary strategies that may improve maternal nutritional status or fetal health. Furthermore, nutritional management may be complicated in patients with IBD because persistent intestinal inflammation and malabsorption can contribute to impaired nutritional status [17,19]. These knowledge gaps highlight the need to better characterize the role of nutrition in pregnancies complicated by IBD.

Pregnant women with IBD are at increased risk for adverse maternal and neonatal outcomes [9,10,16,18,20]. Chronic inflammation and impaired nutrient absorption may further contribute to nutritional deficiencies in this population [19]. Despite growing recognition of these risks, evidence-based dietary recommendations tailored to pregnant women with IBD remain limited. This lack of guidance underscores the need to better understand the relationship between maternal nutrition, IBD, and fetal health to inform future clinical practice and research.

Objective and review question

Although maternal nutrition is recognized as an important determinant of pregnancy outcomes, its role in pregnancies complicated by IBD remains poorly understood. Existing studies have primarily focused on disease activity, medication safety, and obstetric outcomes rather than nutrition-specific factors that may influence maternal nutritional status and fetal health. Therefore, a scoping review was conducted to synthesize the available evidence, identify gaps in the literature, and inform priorities for future research and clinical care. A preliminary search of MEDLINE, the Cochrane Database of Systematic Reviews, and JBI Evidence Synthesis was conducted, and no current or underway systematic reviews or scoping reviews on the topic were identified. This review attempted to answer the question of “What role does nutrition play in fetal development in pregnant women with IGDs?”

## Review

Methodology

Search Strategy Details

We conducted a scoping review of the literature using the electronic databases Embase, Web of Science, Ovid MEDLINE, and CINAHL. These databases were selected to ensure broad coverage of peer-reviewed literature relevant to the review question. All databases were searched on September 10, 2024. The search strategy was developed by the review team in consultation with a medical librarian and incorporated both controlled vocabulary and free-text terms related to IBD, pregnancy, fetal development, nutritional deficiency, dietary restriction, and vitamin supplementation. These concepts were selected to balance search sensitivity with specificity for identifying studies addressing the role of nutrition in fetal development among pregnant women with IGDs. The complete search for each database is provided in the Appendix.

Inclusion and Exclusion Criteria

Our inclusion criteria required studies to be peer-reviewed, published within the last ten years, written in English, from any country, focusing on pregnant patients at any trimester, postpartum patients, and fetuses in the context of nutrition and IGDs. Our exclusion criteria incorporated studies older than ten years, written in languages other than English, case studies, and reviews incorporating patients unable to provide informed consent, multi-fetus pregnancies, or patients with active infection, including human immunodeficiency virus.

Types of Sources

This scoping review evaluated both experimental and quasi-experimental study designs, including randomized controlled trials, non-randomized controlled trials, before-and-after studies, and interrupted time-series studies. In addition, analytical observational studies, including prospective and retrospective cohort studies, case-control studies, and analytical cross-sectional studies, were considered for inclusion.

Study/Source of Evidence Selection

Following the search, all identified citations were collated and uploaded into EndNote, and duplicates were removed. Titles and abstracts were then screened by independent reviewers for assessment against the inclusion criteria for the review. Potentially relevant sources were retrieved in full, and their citation details reported. The full text of selected citations was assessed in detail against the inclusion criteria by at least two independent reviewers. Reasons for exclusion of sources of evidence at full text that do not meet the inclusion criteria were recorded and reported in the scoping review. Any disagreements that arose between the reviewers at each stage of the selection process were resolved through discussion or with an additional reviewer/s. The results of the search and the study inclusion process were reported in full in the final scoping review and presented in a Preferred Reporting Items for Systematic Reviews and Meta-Analyses (PRISMA) extension for scoping review (PRISMA-ScR) flow diagram.

Data Management

Records and data were managed throughout the review with utilization of Rayyan (Rayyan Systems, Inc., Cambridge, MA) and Microsoft Excel/Google Sheets (Microsoft Corp., Redmond, WA) to separate and evaluate the search terms from each database.

Data Extraction

First, titles and abstracts were evaluated to assess their relevance to the study objectives using Rayyan.ai. Subsequently, the potentially relevant articles were first reviewed by abstract through the artificial intelligence (AI) program. Therefore, AI was primarily used as an initial screening tool and organizational tool in our study. Papers with no immediately obvious exclusion criteria underwent further review to determine their eligibility for inclusion. The selection process was conducted independently by two researchers, and any discrepancies were resolved through consensus or consultation with a third researcher when necessary. Studies that met the inclusion criteria were further subjected to data extraction. Data were extracted from papers included in the scoping review by two independent reviewers using a data extraction tool developed by the reviewers in Excel. The data extracted included specific details about the participants, concept, context, study methods, and key findings relevant to the review question.

Critical Appraisal of Individual Sources of Evidence

Sources were analyzed on November 11, 2024, based on the size and demographics of participant groups, participant recruitment, and study quality. A variety of databases such as Embase, MEDLINE, Web of Science, and CINAHL were utilized to gather the sources. The process was made more transparent by predetermining the objective and methods. Each study was independently assessed by the primary and secondary reviewers, using the Joanna Briggs Institute (JBI ) critical appraisal checklists that were relevant for the study type, as identified per publication. This appraisal instrument comprises a checklist of 11 questions from JBI that were answered as “yes”, “no”, or “unclear” to guide the appraisal process and provide an effective evaluation of the quality of an article. This checklist allows for a more concise way of acknowledging the amount of bias in each paper, given the classification of high risk, moderate risk, or low risk. Two researchers read all semi-finalist articles in depth and scored each article with the JBI checklist. Studies meeting the predefined eligibility criteria were critically appraised using the appropriate JBI critical appraisal checklist for each study design. Critical appraisal was used to characterize methodological quality and potential risk of bias among the included evidence rather than as a standalone measure of study relevance. The appraisal findings were considered when interpreting the strength and limitations of the available evidence. The purpose of this appraisal was to determine the quality of the results, relevance, and bias in the review, as well as to determine potential limitations and bias within the literature itself. Our search and selection processes are identified in the PRISMA flowchart (Figure 1).

**Figure 1:**
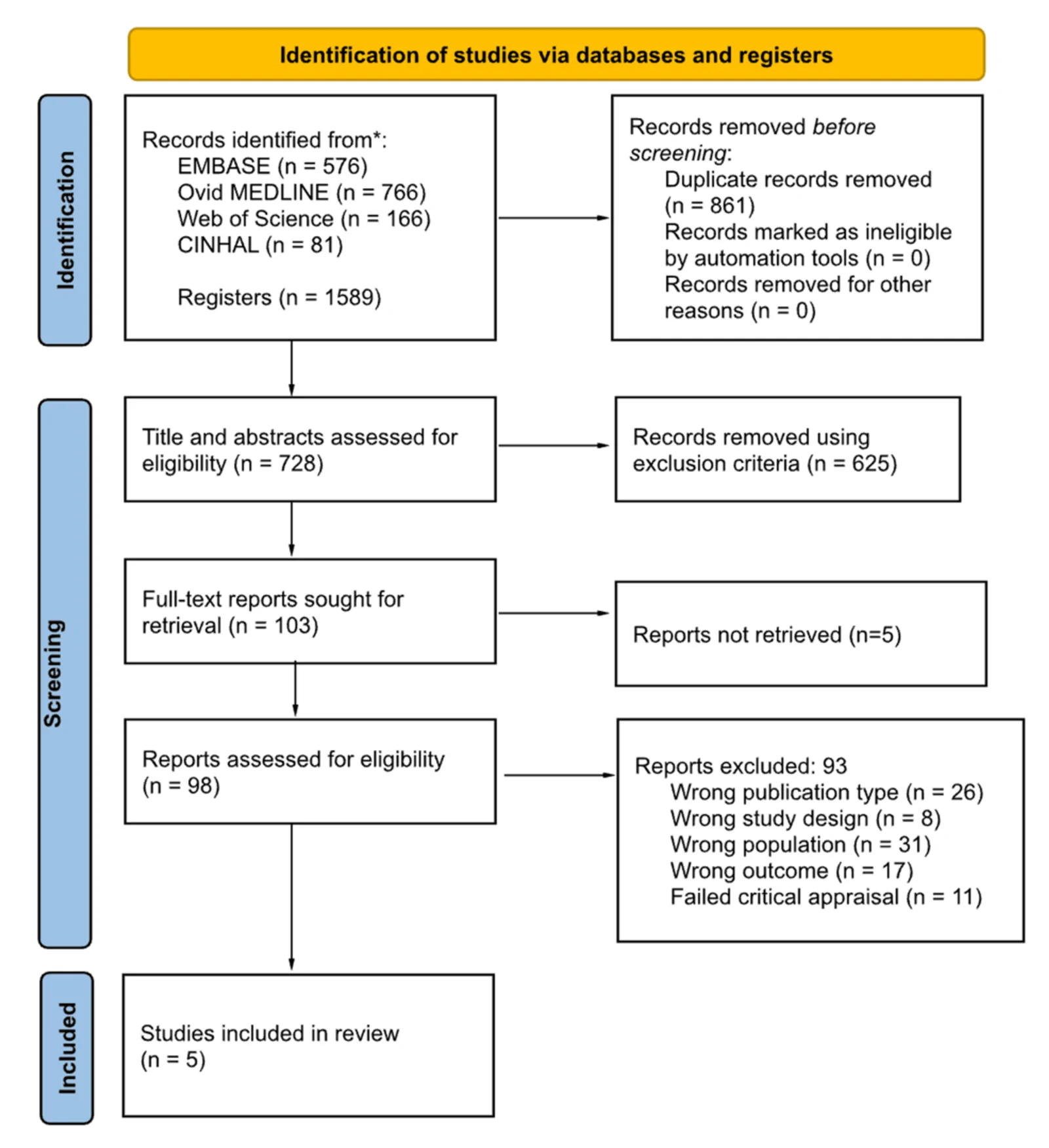
PRISMA flow diagram of the search and selection process. PRISMA, Preferred Reporting Items for Systematic Reviews and Meta-Analyses

Results

Overview

The systematic search identified 1,589 records across four electronic databases. After removal of 861 duplicates, 728 records underwent title and abstract screening. A total of 103 full-text articles were assessed for eligibility, of which 98 were retrieved for review. Following application of the predefined inclusion and exclusion criteria and critical appraisal, five studies met all eligibility criteria and were included in this scoping review (Figure 1). The primary reasons for exclusion included incorrect publication type, study design, study population, or reported outcomes.

Characteristics of Included Studies

The five included studies were published between 2018 and 2023 and consisted primarily of prospective study designs, including analytical cohort, observational, questionnaire-based, and interventional studies [21-25]. Three studies originated from the IBD MOM multidisciplinary clinic at Shaare Zedek Medical Center in Jerusalem, Israel [21-23], while one study was conducted in Norway [24] and one in the United States [25]. Sample sizes varied considerably across studies, ranging from 68 participants in a prospective dietary assessment study [25] to more than 68,000 participants in a large population-based cohort study [24], reflecting the diversity of study designs included in this review. Collectively, the included studies evaluated maternal nutritional intake [24,25], multidisciplinary antenatal care [21,23], medication exposure during pregnancy [22], and maternal and fetal outcomes among women with IBD [21-25]. Overall, the studies demonstrated substantial heterogeneity in study design, participant populations, outcome measures, and methods of nutritional assessment, limiting direct comparison across studies.

IBD MOM Multidisciplinary Clinic

Three included studies were conducted at the IBD MOM multidisciplinary clinic at Shaare Zedek Medical Center in Jerusalem, Israel [21-23]. The clinic provides specialized care for pregnant women with IBD, with data collected on disease activity, medication use, and pregnancy outcomes [21-23]. Because these studies were conducted at the same specialized referral center during overlapping enrollment periods, potential overlap in participant populations could not be excluded. This concentration of evidence from a single referral population was considered when interpreting the findings and their generalizability.

Nutritional Intake

Two studies explored the impact of intake in pregnant patients with IBD, relating to the intake of either dietary nutrients recommended for pregnant patients or the intake of dairy products in pregnant patients with IBD [24,25]. These studies took place in different geographic locations and focused on different elements of dietary intake; however, they related findings to fetal outcomes (Table 1). Olendzki et al. noted in the prospective intervention trial the nutritional deficiencies in all pregnant patients due to inadequate dietary intake. Olendzki et al. identified inadequate dietary intake among pregnant women both with and without IBD, with lower adherence to dietary guidelines among women with IBD. The authors highlighted the potential implications of inadequate maternal dietary intake and chronic inflammation for maternal and infant health [25]. Bengston et al. focused on analyzing the intake of dairy products and the proportion of protein in dairy products in both pregnant patients with IBD and pregnant patients without IBD and the association with smaller gestational age infants. Women with IBD reported lower dairy intake and a lower proportion of protein from dairy sources than women without IBD. Contrary to the authors’ hypothesis, lower dairy intake among women with IBD was associated with a reduced risk of delivering an infant who was small for gestational age (SGA) [24]. They associated this relevant finding with the assumption that many patients with IBD experience lactose malabsorption; therefore, the diarrheal response may impact fetal growth in pregnant patients with IBD [24]. Both studies analyzed patients with inflammatory gut disorders and control patients over time; however, some limitations were present and acknowledged in both, including methodological limitations due to recall bias, self-report bias, and environment (Table 1).

**Table 1:** Summary table of included studies. IBD, inflammatory bowel disease; POIBD, pregnancy-onset inflammatory bowel disease; IBD-MOM, inflammatory bowel disease mothers; 6-TGN, 6-thioguanine nucleotide; CC, community clinic; CD, Crohn disease; UC, ulcerative colitis; TNF, tumor necrosis factor

Author, Year	Aim of Study	Study setting and participants’ details	Methods	Key findings, results, and conclusions	Limitations
Bengston et al. (2020) [24]	To examine the impact of the proportion of protein from dairy sources on inadequate gestational weight gain and small-for-gestational-age infants in IBD and non-IBD mothers.	Pregnant women from Norway from 1999 to 2008, including 148 with Crohn disease, 194 with ulcerative colitis, and 68,858 non-IBD mothers.	Participants answered comprehensive questionnaires assessing diet throughout pregnancy. Logistic regression analyses were used to model multivariate associations.	Intake of dairy products was lower in IBD mothers than in non-IBD mothers. Lower intake of dairy products in IBD mothers was associated with a reduced risk of small-for-gestational-age infants compared with non-IBD mothers and IBD mothers with a high proportion of protein from dairy sources.	The questionnaires used resulted in recall bias in obtaining dietary information, a lack of information regarding disease activity, and concurrent use of IBD drugs. IBD mothers’ disease activity was not followed using clinical examination and biomarkers.
Koslowsky et al. (2018) [21]	The aim of the study was to evaluate the disease and pregnancy courses and outcomes of patients with pregnancy-onset IBD (POIBD) in a single referral center.	237 patients, 31 (15%) POIBD and 206 (85%) non-POIBD, were included.	A prospective analytical study of all pregnant IBD patients treated at the IBD-MOM clinic between 2011 and 2016. Pregnant women with a first diagnosis of IBD during pregnancy, POIBD, were compared with those with an antepregnancy diagnosis of IBD, non-POIBD.	The maternal outcome was shown to be better in the POIBD group. Eight early spontaneous pregnancy losses occurred, all in the non-POIBD group. No POIBD patient underwent cesarean section, compared with 42 (21%) in the non-POIBD group. The mean birth weight and gestational age at delivery were similar between the groups. Rates of prematurity and low birth weight were similar between POIBD and non-POIBD patients. Apgar scores below 7 at 5 minutes and the need for intensive care admission were seen in only 1 patient from the non-POIBD group.	The women’s ages in this study differed between the groups, which may reflect sampling bias. Since more than half of the patients were diagnosed during the first and second trimesters and were under strict surveillance, they may have reached a controlled disease state and consequently had a better newborn outcome. Another limitation is the limited number of patients included, which does not allow full analyses of the many confounders. Many non-POIBD patients were prospectively followed only from pregnancy and not from diagnosis.
Koslowsky et al. (2019) [22]	To evaluate whether 6-TGN exposure during pregnancy in patients with IBD influences anemia in infants at 1 year of life.	Pregnant women with IBD between 2009 and 2015.	A comparative observational study that monitored hemoglobin levels and signs of anemia in infants aged 9 to 15 months born to women exposed to thiopurines compared with infants without maternal thiopurine exposure. Data were collected from clinical files and questionnaires.	Thiopurine/6-TGN exposure during pregnancy was not associated with an increased risk of anemia in infants. Thiopurine therapy during pregnancy is safe for neonates.	The study explored only 34 mothers who were exposed to recall bias. The study failed to retrieve infant nutritional status data.
Olendzki et al. (2023) [25]	To examine the diet quality and dietary intake of pregnant women with and without IBD.	Study participants were recruited by clinical research coordinators in outpatient gastrointestinal clinics and via online advertisements. 68 pregnant women with IBD and 65 without were assessed.	Three 24-hour dietary recalls during 27 to 29 weeks of gestation, along with pre- and probiotic food frequency questionnaires, were utilized for data collection.	Most women in this study did not reach the recommended dietary nutrient goals, which is especially concerning for those with IBD. Dietary guideline goals were significantly lower in women with IBD.	The nature of this study introduced self-report bias, under- and overestimation, and memory bias. Additionally, participant data regarding medication, supplements, or IBD activity status were not considered. This study was conducted during the pandemic, which may have affected the data.
Shitrit et al. (2018) [23]	To evaluate and compare the outcomes of perinatal IBD-MOM clinic patients and antenatal and gastrointestinal disease community clinic patients.	A prospective study of 90 women in the IBD-MOM clinic and 206 in the IBD CC from 2011 to 2015.	The IBD-MOM Disease Severity Score was used to analyze active disease around conception, exacerbations during pregnancy, the use of medications before or during pregnancy, IBD-related hospitalizations, and active perianal disease for CD or active proctitis for UC.	Women in the IBD-MOM group were more likely to receive steroids and anti-TNF agents, be nulliparous, and have active disease during labor or postpartum. Women with IBD were more likely to have cesarean sections. Those with moderate to severe IBD benefited from the multidisciplinary clinic.	This study did not assess the benefit of this care for those with less severe IBD. Further validation of the IBD-MOM Disease Severity Score is needed.

Medication Use

One study explored how medication intake in pregnant mothers with IBD can affect fetal outcomes. Thiopurine therapy is an efficacious treatment for IBD and is teratogenically safe to use during pregnancy to keep the mother in remission [22]. The study investigated the correlation between thiopurine metabolite exposure, specifically 6-thioguanine nucleotides (6-TGN), and infant anemia within the first year [22]. Data were collected from clinical files and maternal questionnaires, as well as infant blood test results (Table 1). The study concluded that 6-TGN exposure during pregnancy was not associated with a higher rate of neonatal anemia [22]. Limitations of the study were that it was conducted in a small population and that the questionnaire used for data collection subjected the mothers to recall bias. Data on infant nutritional status were also not collected (Table 1).

*Multidisciplinary Antenatal Care* 

Two studies explored the antenatal diagnosis and care of pregnant mothers and the subsequent fetal outcomes. Shitrit et al. investigated the differences in perinatal outcomes between mothers who received antenatal care at the IBD MOM clinic and mothers who received antenatal care at gastrointestinal disease community clinics (IBD CC), and later compared the two groups to healthy pregnant women to better characterize perinatal risks for pregnant mothers with IBD [23]. The IBD MOM clinic is a multidisciplinary, single-center clinic established to benefit mothers with IBD and their neonates [23]. Using a non-validated IBD MOM disease severity score, it was concluded that mothers who received antenatal care at the IBD MOM clinic had a significantly higher disease severity score and overall preterm delivery rate than IBD CC mothers [23]. Comparing both IBD MOM and IBD CC mothers to healthy pregnant women, IBD MOM and IBD CC mothers were more likely to have cesarean sections and delivered babies with significantly lower mean birth weight [23]. This study did not assess the benefit of this care for those with less severe IBD. Further validation of the IBD MOM Disease Severity Score is needed as well (Table 1). Koslowsky et al. investigated the antenatal diagnosis of pregnancy-onset IBD (POIBD) and the subsequent perinatal outcomes [21]. A prospective analytical study was conducted at the IBD MOM clinic comparing POIBD mothers to non-POIBD mothers. Ultimately, cesarean section did not occur in the POIBD group, and premature birth rates and mean birth weight were similar between the groups [21]. A limitation of this study is that only a limited number of patients were included and that the non-POIBD mothers were followed prospectively from pregnancy, not diagnosis [21].

Studies were grouped in a table by author and year of publication, aim of study, setting, participant details, and methods to efficiently compare and analyze patterns and themes across studies. The articles were then assessed for key findings, conclusions, and limitations, which were added to the final summary (Table 1).

Discussion

This scoping review synthesized the available evidence regarding the role of nutrition in fetal outcomes among pregnant women with IBD. The limited number of studies directly evaluating nutritional intake demonstrates that current evidence regarding nutrition during pregnancy in women with IBD remains sparse, with most available literature focusing instead on disease management, medication safety, and multidisciplinary models of care. Although the included studies suggest that multidisciplinary antenatal care, maintenance of disease remission, and attention to maternal nutritional status may be associated with improved maternal and neonatal outcomes, the current evidence remains limited. Only two studies directly evaluated dietary intake; no included study investigated the effects of a nutritional intervention on fetal outcomes, and all included studies were observational in design [21-25]. Therefore, these findings should be interpreted as hypothesis-generating rather than establishing evidence-based clinical recommendations. Although only two included studies directly evaluated dietary intake, the remaining studies were included because they addressed clinical factors closely linked to maternal nutritional status in IBD, including disease remission, medication safety, and multidisciplinary antenatal care. Active intestinal inflammation can impair nutrient absorption, while maintenance of remission through appropriate medical therapy and coordinated prenatal care may indirectly support maternal nutritional status and subsequent pregnancy outcomes. Therefore, these studies provide important contextual evidence regarding the broader clinical environment in which nutritional status influences maternal and fetal health, despite not evaluating dietary interventions directly.

Although deficiencies in micronutrients such as folate, vitamin B12, iron, and magnesium are well-established contributors to adverse pregnancy outcomes in the general obstetric population, none of the studies included in this review directly evaluated these deficiencies among pregnant women with IBD. This highlights an important gap in the current literature and demonstrates that recommendations regarding micronutrient management in this population remain largely extrapolated from broader obstetric evidence rather than disease-specific research.

Perceived Benefits of Nutrition

Current evidence suggests that maternal nutritional status should be considered within the broader context of IBD disease control during pregnancy. Although inadequate dietary intake has been identified among pregnant women with IBD, the available literature does not establish that nutritional optimization or specific dietary modifications directly improve fetal outcomes [24,25]. Rather, the findings suggest that maintenance of disease remission, appropriate medical therapy, and multidisciplinary antenatal care may indirectly support maternal nutritional status and favorable pregnancy outcomes [21-23].

Importantly, associations observed in individual studies should not be interpreted as evidence supporting specific dietary recommendations. For example, the observed association between lower dairy intake and a reduced risk of small-for-gestational-age infants does not establish a causal benefit of dairy restriction [24]. Overall, these findings support further investigation of nutrition as a component of comprehensive IBD management during pregnancy, particularly through prospective studies evaluating specific nutritional interventions.

Benefits and Risks of Implementation

Although the available evidence does not support specific nutritional interventions, multidisciplinary prenatal care may provide an opportunity to identify and address disease activity, nutritional deficiencies, and dietary concerns in pregnant women with IBD. Individualized nutritional assessment and counseling may therefore be reasonable components of comprehensive prenatal management. However, recommendations regarding specific dietary restrictions or supplementation should be individualized and based on established clinical indications until prospective studies demonstrate a benefit in this population. Implementation of multidisciplinary care may also be limited by insurance coverage, access to specialists, and healthcare costs.

Strengths

Despite the limited number of eligible studies, this review has several strengths. A comprehensive search strategy across four major databases was used in accordance with PRISMA-ScR recommendations. Independent screening, predefined eligibility criteria, and critical appraisal of included studies improved methodological rigor. By synthesizing the currently available evidence, this review identifies important gaps in the literature and highlights priorities for future research regarding nutritional management during pregnancy in women with IBD.

Limitations

Overall, the methodological quality of the included studies was poor. The populations assessed and data collected in each of the five studies varied significantly. The use of self-administered questionnaires and surveys in three studies introduces the potential for recall and memory bias, leading to decreased reliability of the data collected. Furthermore, studies either limited their populations by assessing only severe cases of IBD among pregnant women or did not differentiate disease severity at all. Several studies failed to collect data on clinical examination findings, biomarkers, infant nutritional status, and medication intake. With the application of our inclusion and exclusion criteria to the results of the included databases, we identified a total of five papers for inclusion in this review, a surprisingly small number given the growing prevalence and severity of IBD among women. We believe this may be due to the restrictions and controversy surrounding research involving pregnant subjects. Furthermore, the included studies primarily reported pregnancy and neonatal outcomes rather than direct measures of fetal development. Consequently, outcomes such as birth weight, gestational age, preterm birth, and neonatal anemia should be interpreted as indirect indicators of fetal health.

Although the search strategy was developed in consultation with a medical librarian, it emphasized nutrition-related concepts, including nutritional deficiency, dietary restriction, and vitamin supplementation. Broader terms such as maternal nutrition, micronutrients, dietary interventions, fetal growth, birth outcomes, and neonatal outcomes were not explicitly incorporated into the search strategy. While this approach was intended to improve specificity for the review question, it may have limited the retrieval of additional relevant studies and contributed to the relatively small number of eligible articles identified.

Despite the small number of included studies, the use of multiple databases, reference-list screening, and independent study selection strengthened the comprehensiveness of the review. Nevertheless, relevant studies may have been missed because of limitations in the search terminology and predefined eligibility criteria. A lack of demographic diversity must be considered, as the locations of these studies represented populations from only three countries: Israel, Norway, and the United States.

Additionally, three of the five included studies originated from the same multidisciplinary IBD MOM clinic in Jerusalem, Israel. Although these studies addressed different research questions, they were conducted within the same specialized referral center during overlapping time periods, raising the possibility of overlapping patient populations. Because participant-level overlap could not be confirmed or excluded based on the published reports, the findings from these studies should not be interpreted as three entirely independent sources of evidence. Furthermore, the concentration of evidence from a single tertiary referral center may limit the external validity of the review, as patient demographics, disease severity, referral patterns, access to multidisciplinary care, and healthcare system characteristics may differ substantially from those in other settings.

Indications for Future Research

Throughout all phases of our review process, we recognized the limited body of research available on nutrition and fetal outcomes in pregnancies complicated by IBD. Although micronutrients such as folate, vitamin B12, iron, magnesium, and vitamin D are well established as important determinants of healthy pregnancy outcomes, none of the studies included in this review directly evaluated maternal micronutrient deficiencies or their association with fetal development in women with IBD. Future research should therefore directly investigate micronutrient status in pregnant women with IBD and determine how deficiencies influence fetal growth, neonatal outcomes, and long-term child health. Additional research on IBD biomarker levels in both pregnant mothers and their neonates, the pathology of POIBD, dietary patterns during pregnancy, and long-term follow-up of offspring would further improve understanding of fetal outcomes in this population. Future studies should also address the limitations of the current literature, including the need for larger, adequately powered, prospective longitudinal studies. Finally, research should be expanded to include a broader range of IGDs, as no eligible studies evaluating other IGDs, such as celiac disease, met the inclusion criteria for this review, highlighting an important gap in the literature.

Future evidence syntheses may benefit from expanding the search terminology to include additional nutrition- and pregnancy-related concepts, such as maternal nutrition, micronutrients, dietary interventions, fetal growth, birth outcomes, and neonatal outcomes, to ensure comprehensive capture of the available literature.

## Conclusions

This scoping review examined the available literature regarding the role of nutrition in fetal development among pregnant women with IGDs. The included studies suggest that multidisciplinary antenatal care, maintenance of disease remission, and attention to maternal nutritional status may contribute to improved maternal and neonatal outcomes. However, the available evidence remains limited, consisting primarily of observational studies, with only two studies directly evaluating dietary intake and none assessing the effects of nutritional interventions on fetal outcomes. Consequently, the current evidence is insufficient to support specific nutritional recommendations for clinical practice. Future prospective studies evaluating dietary interventions, maternal nutritional deficiencies, and long-term fetal outcomes are needed to better inform evidence-based nutritional management during pregnancy among women with IGDs.

## Appendices

Appendix 

**Table 2:** Search queries for Embase, Ovid Medline, Web of Science, and Cinahl.

No.	Search Query Embase (09/10/2024)	Results
#1	'inflammatory bowel disease'/exp	221053
#2	("inflammatory bowel disease" OR "inflammatory bowel diseases" OR "crohn disease" OR "crohns disease" OR "ulcerative colitis" OR "inflammatory gut disease" OR "inflammatory gut diseases"):ab,ti,kw	170172
#3	#1 OR #2	253556
#4	'pregnancy'/exp	917699
#5	('child bearing' OR 'childbearing' OR 'gestation*' OR 'gravidity' OR 'intrauterine pregnan*' OR 'labor presentation' OR 'labour presentation' OR 'pregnan* maintenance' OR 'pregnan* trimesters' OR 'pregnan*'):ab,ti,kw	798584
#6	#4 OR #5	1200568
#7	'fetus development'/exp	33421
#8	('fetus develop*' OR 'fetal develop*' OR 'foetal develop*' OR 'foetus develop*'):ab,ti,kw	17128
#9	#7 OR #8	42065
#10	'nutritional deficiency'/exp	339905
#11	('alimentary deficien*' OR 'defective diet' OR 'deficien* disease' OR 'deficien* diseases' OR 'deficien*, nutritional' OR 'deficien* diet' OR 'diet deficien*' OR 'diet insufficien*' OR 'diet, defective' OR 'dietary deficien*' OR 'dietary insufficien*' OR 'food deficien*' OR 'micronutrient deficien*' OR 'micronutrient insufficien*' OR 'nutrition deficien*' OR 'nutrition* deficit' OR 'nutritive deficien*' OR 'nutrition* deficien*' OR 'vitamin deficien*'):ab,ti,kw	30982
#12	#10 OR #11	352118
#13	'diet restriction'/exp	174472
#14	('dietary restriction' OR 'food restriction' OR 'restricted diet' OR 'restrictive diet' OR 'diet restriction'):ab,ti,kw	16624
#15	#13 OR #14	179895
#16	'vitamin supplementation'/exp	41874
#17	#3 AND #6 AND #12	250
#18	#3 AND #9	99
#19	#15 OR #16	220640
#20	#3 AND #6 AND #19	63
#21	#9 AND #12 AND #19	148
#22	#17 OR #18 OR #20 OR #21	530
No.	Search Query Ovid Medline (09/10/2024)	Results
#1	exp Inflammatory Bowel Diseases/	102645
#2	("inflammatory bowel disease" or "inflammatory bowel diseases" or "crohn disease" or "crohns disease" or "ulcerative colitis" or "inflammatory gut disease" or "inflammatory gut diseases").ab,ti,kf.	130547
#3	exp Pregnancy/	1040578
#4	('child bearing' or 'childbearing' or 'gestation*' or 'gravidity' or 'intrauterine pregnan*' or 'labor presentation' or 'labour presentation' or 'pregnan* maintenance' or 'pregnan* trimesters' or 'pregnan*').ab,ti,kf.	777994
#5	exp Nutrition Disorders/	445065
#6	('alimentary deficien*' or 'defective diet' or 'deficien* disease' or 'deficien* diseases' or 'deficien*, nutritional' or 'deficien* diet' or 'diet deficien*' or 'diet insufficien*' or 'diet, defective' or 'dietary deficien*' or 'dietary insufficien*' or 'food deficien*' or 'micronutrient deficien*' or 'micronutrient insufficien*' or 'nutrition deficien*' or 'nutrition* deficit' or 'nutritive deficien*' or 'nutrition* deficien*' or 'vitamin deficien*').ab,ti,kf.	24330
#7	exp Fetal Development/	104705
#8	('fetus develop*' or 'fetal develop*' or 'foetal develop*' or 'foetus develop*').ab,ti,kf.	13394
#9	exp Diet/	343464
#10	('dietary restriction' or 'food restriction' or 'restricted diet' or 'restrictive diet' or 'diet restriction').ab,ti,kf.	12350
#11	exp Dietary Supplements/	108267
#12	1 or 2	145850
#13	3 or 4	1262232
#14	5 or 6	458958
#15	7 or 8	114933
#16	9 or 10 or 11	438714
#17	12 and 13 and 14	55
#18	12 and 15	82
#19	12 and 13 and 16	74
#20	14 and 15 and 16	589
#21	13 and 20	568
#22	17 or 18 or 19 or 21	764
No.	Search Query Web of Science (09/10/2024)	Results
1	TS=(“inflammatory bowel disease” OR “inflammatory bowel diseases” OR “crohn disease” OR “crohns disease” OR “ulcerative colitis” OR “inflammatory gut disease” OR “inflammatory gut diseases”)	161976
2	TS=(“child bearing” OR “childbearing” OR “gestation” OR “gravidity” OR “intrauterine pregnancy” OR “labor presentation” OR “labour presentation” OR “pregnancy maintenance” OR “pregnancy trimesters” OR “pregnancy”)	617435
3	TS=(“fetus development” OR “fetal development” OR “foetal development” OR “foetus development”)	13835
4	TS=(“alimentary deficien*” OR “defective diet” OR “deficien* disease” OR “deficien* diseases” OR “deficien*, nutritional” OR “deficien* diet” OR “diet deficien*” OR “diet insufficien*” OR “diet, defective” OR “dietary deficien*” OR “dietary insufficien*” OR “food deficien*” OR “micronutrient deficien*” OR “micronutrient insufficien*” OR “nutrition deficien*” OR “nutrition* deficit” OR “nutritive deficien*” OR “nutritional deficien*” OR “vitamin deficien*”)	23652
5	TS=(“diet influence” OR “diet regimen” OR “diet surveys” OR “dietary effect” OR “dietary influence” OR “dietary survey” OR “dietary surveys” OR “dieting” OR “diet”)	611971
6	#1 AND #3	29
7	#3 AND #4	68
8	#2 AND #3 AND #4	51
9	#1 AND #2 AND #5	69
10	#6 OR #7 OR #8 OR #9	165
No.	Search Query Cinahl (09/10/2024)	Results
S1	(MH "Inflammatory Bowel Diseases+") OR "inflammatory bowel disease"	23725
S2	("inflammatory bowel disease" OR "inflammatory bowel diseases" OR "crohn disease" OR "crohns disease" OR "ulcerative colitis" OR "inflammatory gut disease" OR "inflammatory gut diseases"):ab,ti,kw	8945
S3	S1 OR S2	25020
S4	(MH "Pregnancy+") OR "pregnancy"	290894
S5	('child bearing' OR 'childbearing' OR 'gestation*' OR 'gravidity' OR 'intrauterine pregnan*' OR 'labor presentation' OR 'labour presentation' OR 'pregnan* maintenance' OR 'pregnan* trimesters' OR 'pregnan*'):ab,ti,kw	9498
S6	S4 OR S5	291099
S7	(MH "Fetal Development+") OR "fetus development"	32578
S8	('fetus develop*' OR 'fetal develop*' OR 'foetal develop*' OR 'foetus develop*'):ab,ti,kw	17282
S9	S7 OR S8	32678
S10	(MH "Malnutrition+") OR "nutritional deficiency"	13851
S11	('alimentary deficien*' OR 'defective diet' OR 'deficien* disease' OR 'deficien* diseases' OR 'deficien*, nutritional' OR 'deficien* diet' OR 'diet deficien*' OR 'diet insufficien*' OR 'diet, defective' OR 'dietary deficien*' OR 'dietary insufficien*' OR 'food deficien*' OR 'micronutrient deficien*' OR 'micronutrient insufficien*' OR 'nutrition deficien*' OR 'nutrition* deficit' OR 'nutritive deficien*' OR 'nutrition* deficien*' OR 'vitamin deficien*'):ab,ti,kw	0
S12	S10 OR S11	13878
S13	(MH "Restricted Diet+") OR "diet restriction"	14542
S14	('dietary restriction' OR 'food restriction' OR 'restricted diet' OR 'restrictive diet' OR 'diet restriction'):ab,ti,kw	1552
S15	S13 OR S14	14564
S16	(MH "Dietary Supplements+") OR "dietary supplementation"	59852
S17	S3 AND S6 AND S12	2
S18	S3 AND S9	41
S19	S15 OR S16	73576
S20	S3 AND S6 AND S19	20
S21	S9 AND S12 AND S19	21
S22	S17 OR S18 OR S20 OR S21	81
